# Termites and Chinese agricultural system: applications and advances in integrated termite management and chemical control

**DOI:** 10.1111/1744-7917.12726

**Published:** 2019-10-15

**Authors:** Farhan Ahmad, Hatem Fouad, Shi‐You Liang, Yin Hu, Jian‐Chu Mo

**Affiliations:** ^1^ Ministry of Agriculture Key Lab of Molecular Biology of Crop Pathogens and Insect Pests, Institute of Insect Sciences, College of Agricultural and Biotechnology Zhejiang University Hangzhou China; ^2^ Entomology Section Central Cotton Research Institute, Sakrand Shaheed Benazirabad Sindh Pakistan; ^3^ Department of Field Crop Pests, Plant Protection Research Institute Agricultural Research Centre Cairo Egypt; ^4^ National Termite Control Center Hangzhou China

**Keywords:** entomopathogens, geographical distribution, integrated termite management, termite

## Abstract

Termites are eusocial arthropod decomposers, and improve soil fertility, crop yield, and also are used by humans for their benefits across the world. However, some species of termites are becoming a threat to the farming community as they are directly and indirectly causing major losses to the agricultural system. It is estimated that termites cost the global economy more than 40 billion USD annually, and considerable research has been done on their management. In this review, we present the available information related to sustainable and integrated termite management practices (ITM). Furthermore, we insist that the better management of this menace can be possible through: (i) improving traditional methods to keep termites away from crops; (ii) improving agricultural practices to maintain plants with more vigor and less susceptible to termite attack; and (iii) integration of available techniques to reduce termite infestation in crops and surroundings. The application of an effective combination of traditional practices with recently developed approaches is the best option for agricultural growers. Moreover, keeping in mind the beneficial nature of this pest, more innovative efforts for its management, particularly using rapidly emerging technology (e.g., RNA interference), are needed.

## Introduction

Termites are eusocial small insects (Isoptera: Termitidae), constitute 10% of all animal biomass (van Huis, 2017), and are primarily distributed in the tropical and sub‐tropical regions of the world (Brune, 2014; Bonachela *et al*., 2015). Three thousand species of termites have been described, and only very few are considered to be the agricultural pests (Brune, 2014). Termites originated from Sub‐Saharan Africa, and species numbers are highest in rainforest habitats (Poulsen *et al*., 2014). By their habitat they are categorized as subterranean termites (live beneath the soil), dry wood termites (spend their life in dry wood) and damp wood termites (prefer to live in moist wood) (Govorushko, 2019). However, Brauman and coworkers divided termites into four groups by their feeding behavior: (i) wood feeders; (ii) soil feeders: (iii) fungus growing and (iv) grass feeders (Brauman *et al*., 2015).

Termites are the most dominant arthropod decomposers and have an extraordinary ecological impact on both agricultural and nonagricultural ecosystems through playing a significant role in the global carbon cycle, decomposition process and mineralization of nutrient‐rich cellulose (Traoré *et al*., 2015). Their tunneling behavior improves soil fertility, nutrient availability, water infiltration and crop yield (Evans *et al*., 2011; Lagendijk *et al*., 2016; Govorushko, 2019). Moreover, they are also used by mankind in food and medicine, in superstitious beliefs, arts and literature (van Huis, 2017) across the world.

Despite their beneficial nature, they often cause major losses to humans through damaging anything that particularly consists of cellulose, such as books, stored timbers, wooden structures, buildings, stored grain products, crops, standing trees and forests (Ravan *et al*., 2015). It is estimated that termites cause a noteworthy economic loss of more than 40 billion USD annually worldwide (Rust & Su, 2012; Subekti *et al*., 2015). They damage the agricultural and horticultural crops directly (through feeding on the bark and underlying tissue) and indirectly (the damaged plants become susceptible to pathogenic microbes) resulting in plant death (Paul *et al*., 2018). Their infestation starts from the root and then spreads to the whole plant.

Termites are becoming a threat to the farming community as all major field crops such as sugarcane, cotton, tobacco, cereals (rice, wheat, barley, maize, millet and sorghum), vegetables (tomato, okra, pepper, eggplant, potato and cassava), fruits (guava, citrus, banana, mango, papaya, grapes, mulberry, pineapple, almond, litchi and plum), legumes (beans, cowpea and chickpea), oilseeds (groundnut, sunflower, soybean and sesame) and ornamental plants are affected by termites (Rathour *et al*., 2014; Lin *et al*., 2015).

Although researchers have made considerable progress for developing control strategies (such as cultural, biological, botanical and chemical approaches) globally, the sustainable and integrated management of termites is still a great challenge because they are always hidden in galleries/mounds and it is difficult to reach their living places. Furthermore, integrated pest management (IPM) approaches can be effective if there is proper communication among the stakeholders, all information about the target pest, monitoring of target area, infestation‐based decisions and regular follow‐up to the whole procedure (Li *et al*., 2016).

Keeping in mind the usefulness of termites and their economic importance as pests, in this review, we discuss the available information on integrated and sustainable management practices against termites attacking agricultural system.

## The economic cost and taxonomic diversity of termites: A Chinese perspective

Termites are a real threat to the agricultural system in China. They damage crops, plantations, and forestry from sowing till harvesting, and it is difficult to notice their damage symptoms at an early stage. Their presence in an area causes major problems through infesting both field and store crops, moreover destroying lignocellulosic material. Agronomic crops such as rice, wheat, corn, sorghum, millet, sugarcane, cotton, numerous trees, including camphor tree, Chinese chestnut, eucalyptus, and palm are readily attacked by termites (Li *et al*., 2001), and significant yield losses have been recorded in tropical and sub‐tropical regions. Zhang and Govindaraju (2018) reported termite infestation on sugarcane crops in southern China.

China is very mountainous, and only 15% of the total land available can be cultivated. Most of the arable land is in the band of river valleys and along the southern and eastern coasts. Generally, the soil of cultivated land is acidic and the climate is humid. It is reported that low pH soil and high relative humidity conditions are favorable for better growth of termites (Li *et al*., 2017).

The majority of Chinese farmers irrigate their crops with water from canals, rivers or reservoirs. Termites construct nests and tunnels inside the dams and reservoirs; the result is leaking and collapsing (Zhong & Liu, 2002; Tian *et al*., 2009). More than 90% of dams and reservoirs in southern China are damaged by *Odontotermes formosanus* (Shikraki) and *Macrotermes barneyi* Light (Huang *et al*., 2000). Li and coworkers described that *Odontotermes formosanus* (Shikraki) and *Reticulitermes flaviceps* (Oshima) destroy earthen seawalls of Qiantang River, Zhejiang province, China (Li *et al*., 2017). The termitologists of the country believe that many flood disasters in southern area have been caused by dyke and dam breaks due to termites (Huang *et al*., 2000; Zhong & Liu, 2002). Furthermore, Chinese agriculture is dominated by small‐scale farmers and the majority of them are often unaware of new technologies. Indeed, they are using a number of traditional practices for termite management, but their success is limited.

Termites management and economic losses cost hundreds of million dollars annually. In 2004, the estimated annual economic losses caused 0.3 billion dollars (Zhong & Li, 2004). Forests damage alone by this pest cost approximately 217 million dollars annually (Li *et al*., 2010). The losses in a single province Taiwan run into more than 4 million dollars per annum (Li *et al*., 2011). More than 90% of homes in the south of the Yangtze River are affected by termites (MRP, 2010). Even with management, the damage and economic losses due to termites is increasing steadily. Lenz *et al*. (2003) reported that termites cost around 1 billion dollars annually to the Chinese economy (Table 1).

**Table 1:** Estimates of annual economic losses caused by termites worldwide

Regions	Annual economic losses	References
Australia	$1.5 billion	Staunton, 2012
China (mainland)	$1 billion	Lenz *et al*., 2003
Fiji Islands	$1 million	Chand *et al*., 2018
France	$0.5 billion	Lenz *et al*., 2003
India	$35.12 million	Verma *et al*., 2009
Indonesia	$1 billion	Hadi *et al*., 2016
Japan	$0.8–1 billion	Tsunoda & Yoshumura, 2004
Malaysia	$10–12 million	Yeoh & Lee, 2007
Philippine	$100s million	Acda, 2013
Taiwan China	$4 million	Li *et al*., 2011
Thailand	$0.5 billion	Vongkaluang, 2004
USA	$11 billion	Subekti *et al*., 2015
World	$40 billion	Rust & Su, 2012

**Fig. 1:**
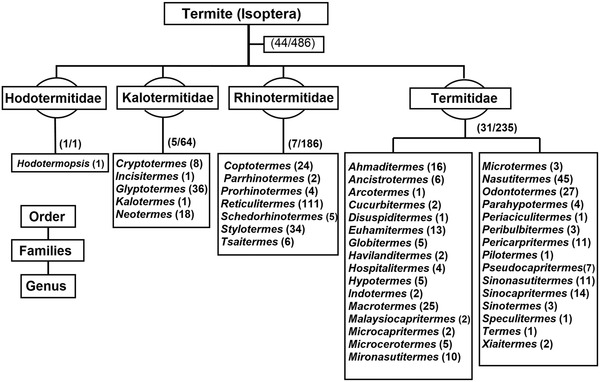
Diagrammatic representation of termite classification in China. (Digits/digits) represent the genera/species and (digits) illustrate the species (Zhong & Li, 2004; Mo *et al*., 2008; Li *et al*., 2010; Lin *et al*., 2015).

**Fig. 2:**
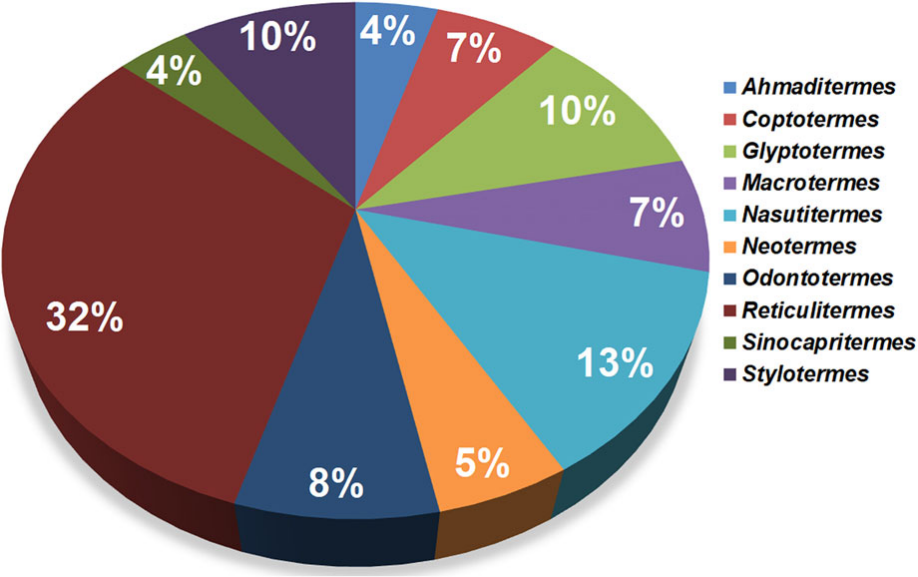
Percentage of termite species numbers of the ten dominant genera in China.

**Fig. 3:**
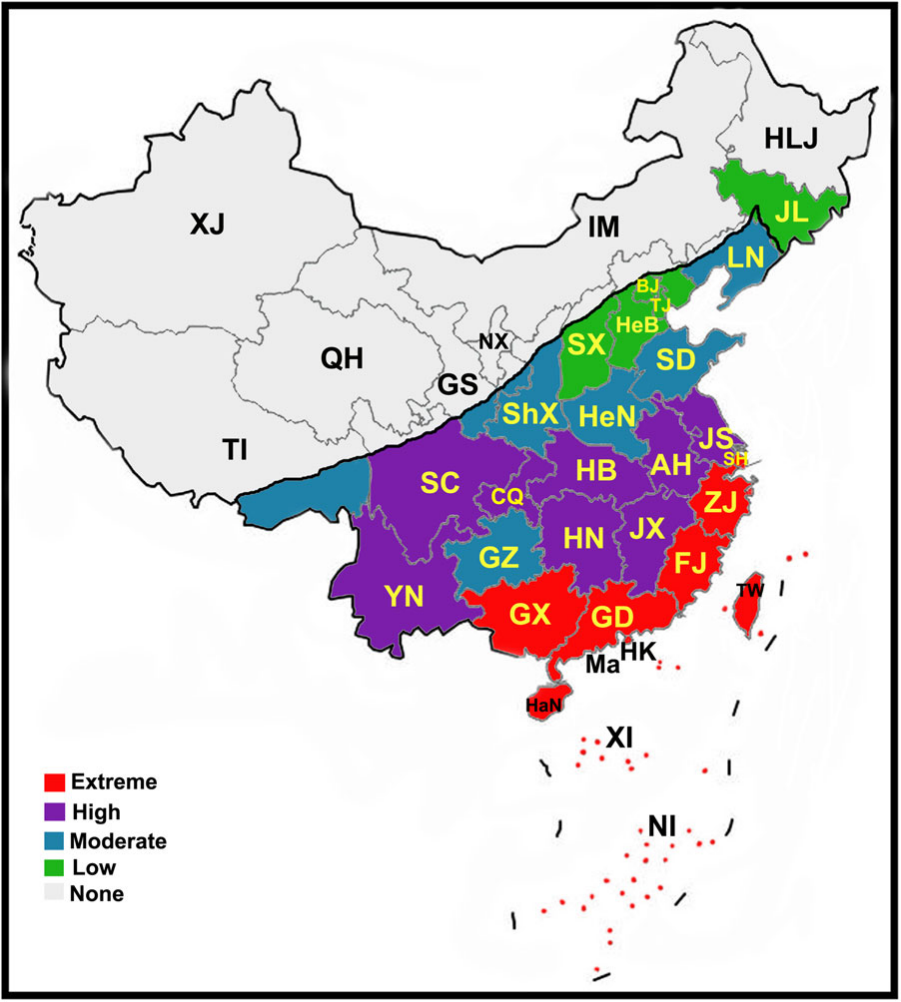
Termite infestation and distribution in China [GS(2019)3200]. Provinces, municipalities and islands are abbreviated as follows: Extreme infestation (GD, Guangdong; GX, Guangxi; HaN, Hainan; FJ, Fujian; ZJ, Zhejiang; SH, Shanghai; Ma, Macau; HK, Hong Kong; TW, Taiwan; XI, Xisha Islands; NI, Nansha Islands). High infestation (HB, Hubei; HN, Hunan; JX, Jiangxi; AH, Anhui; JS, Jiangsu; CQ, Chongqing; SC, Sichuan; YN, Yunnan). Moderate infestation (GZ, Guizhou; ShX, Shaanxi; HeN, Henan; SD, Shandong; LN, Liaoning; some parts of TI, Tibet; some parts of GS, Gansu). Low infestation (BJ, Beijing; TJ, Tianjin; SX, Shanxi; HeB, Hebei; JL, Jilin). No infestation (XJ, Xinjiang; QH, Qinghai; NX, Ningxia; IM, Inner Mongolia; HLJ, Heilongjiang; TI, Tibet; GS, Gansu). Data from Zhong and Li (2004); Mo *et al*. (2008).

Currently, there are over 486 species with 44 genera belonging to Hodotermitidae, Kalotermitidae, Rhinotermitidae and Termitidae families recorded in China (Lin *et al*., 2015). However, *Coptotermes*, *Cryptotermes*, *Glyptotermes*, *Macrotermes*, *Nasutitermes*, *Odontotermes*, *Reticulitermes* and *Stylotermes* are considered to be the most dominant (Figs. 1, 2) (Li *et al*., 2010; Lin *et al*., 2015). The species are distributed mainly in the south, southeast and central regions of the country (Zhong & Li, 2004). Approximately, above ten thousand tons of pesticides have been applied in southern provinces for their infestation suppression. Mo *et al*. (2008) reported that a single province, Yunnan, has more than 125 species of termites. The extremely infested provinces are Chongqing, Fujian, Guangdong, Guangxi, Hainan, Sichuan, Yunnan and Zhejiang (Fig. 3).

## Integrated termite management (ITM)

**Fig. 4:**
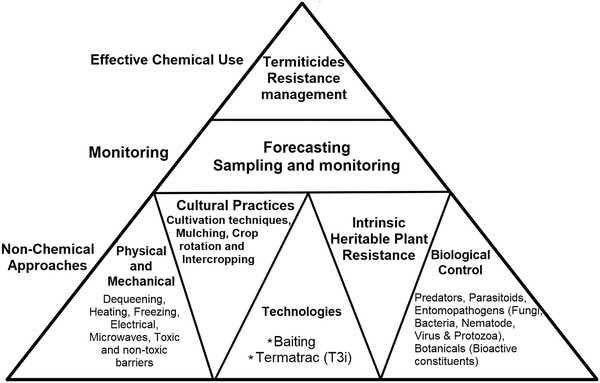
Diagrammatic representation of integrated termite management (ITM) elements.

No doubt, termites are beneficial to humankind and have a tremendous impact on the ecosystem, but we cannot forget their destructive nature as well. They have become a severe problem in the agriculture system worldwide. Therefore, we have to find some possible ways for its effective management. ITM is a sustainable program aiming not to get rid of the termite population but to keep it away or to reduce its activity from economically important areas. ITM can be described as the combination of the available effective control measures that are economically, socially and environmentally safe to humankind (Forschler *et al*., 2007; Forschler, 2011). However, the decision for termite management should be made after getting enough knowledge of termite biology and ecology, infestation level, soil characteristics, cropping systems, cost of control, chemical and non‐chemical termite management approaches and availability of proper termiticides, barriers, baits and professional experts. The pyramid described in Figure 4 represents the principle for termite management. Sustainable termite control is comprised of various strategies, mainly we can categorize as monitoring, chemical and non‐chemical practices. The effective use of termiticides depends on the threshold and resistance information and control actions rely on sampling and monitoring. Further, non‐chemical strategies are an essential component in ITM and techniques include physical and mechanical control (dequeening, heating, freezing, electrical, microwaves, toxic and non‐toxic barriers), cultural control (cultivation techniques, mulching, crop rotation and intercropping), intrinsic heritable plant resistance, biological control (predators, parasitoids, entomopathogens and botanicals), some modern techniques (such as attractive baits and termatrac) and emerging biotechnology tools (e.g., RNA interference). The combination of these strategies such as monitoring and baiting, chemical and physical barriers, baits and biological agents or dust toxicants are regarded as active ITM approaches for termite management. However, the implementation and success of an ITM program are still much needed. Hence, we summarized and synthesized available important information that provides insight into the sustainable management practices regarding termites.

## Physical and mechanical management practices

The management of termites using physical and mechanical means are the common practices worldwide to keep the population at a certain level. These practices comprise of dequeening or removing of the king from the colony, which may destroy the whole colony (Atsbha & Hintsa, 2018). The breaking of termite mounds manually and through tillage also provides a temporary control (Tasida & Gobena, 2013). However, it is difficult to eradicate this pest through this approach as the mound is constructed deep inside the ground and is challenging to reach the target. More labor is required through this means as the mound is made up of hard material and has many dimensions. Further, there may be a possibility of the revival of the colony through substituting their queen.

Extreme high and low temperatures have a negative impact on termite survival (Cao & Su, 2015). The termites are efficiently controlled at high temperatures. Heating and smoke by burning of the crop residues at the mounds to suffocate the colony is a common practice in India (Verma *et al*., 2018) but it is very difficult to penetrate the smoke and heat deep enough to kill reproductive alates as termites have a complex and very sophisticated mound structure. Also, intensive heating makes the soil hydrophobic.

Physical or mechanical barriers are the obstacles used to protect structures from termites. These barriers may be toxic or non‐toxic. The use of chemicals or insecticides (termiticides) in the soil to manage the termite population is an example of a toxin barrier. Manzoor *et al*. (2014) describe that insecticide is very effective when incorporated into the soil. In the case of non‐toxic physical/mechanical barriers, non‐chemical material (such as sand or gravel aggregates, metal mesh or sheeting) are used to prevent termite damage in agricultural crops and commercial buildings (Li *et al*., 2011). A layer of sand instead of soil is applied over the nurseries of fruit trees to prevent termite damage. Li and coworkers conducted a study to investigate the effect of gravel sands as a physical barrier against two termite species *Coptotermes formosanus* and *Reticulitermes flaviceps* under laboratory and field conditions (Li *et al*., 2011). Their results revealed that proper thickness and particle sizes of gravel sands could be used as a barrier material to reduce the invasion of termites (Li *et al*., 2011). Keefer *et al*. (2013) described the effectiveness of aggregate particles as a physical barrier against subterranean termites and reported that all aggregate ratios of particle sizes inhibited tunneling by termites. Tree nurseries can be protected from termites by digging a deep channel around the seedlings which creates a hurdle in developing termite galleries.

Magnets can be used to arrest colony growth. For example, strong bar magnets are placed in the soil near the termite mounds to prevent them from growing. Diba *et al*. (2013) used electromagnetic waves to control subterranean termites *Coptotermes curvignathus* and *C. formosanus*. Maayiem *et al*. (2012) described that application of a mixture of salt and shea butter residues, planting of elephant grass and burying animal material reduced termite outbreak. The use of wood ash near and around crops and termite mounds can be another option in a termite management system. Further, it provides potassium and calcium to the applied area. Atsbha and Hintsa (2018) investigated the impact of wood ash on termite infestation. They used wood ash on the seedling beds of hot pepper and found satisfactory results. However, we can only use this technique in a small area as more labor is required during its application. There is no clear application method and dose rate in the literature. Also, it may be harmful to soil‐beneficial microbes and causes runoff by making soil hydrophobic.

## Cultural management practices

Cultural management practices include clean cultivation, high‐density sowing, soil management, balanced use of fertilizers, proper irrigation, weeding, mulching, timely harvesting, crop rotation and intercropping and so on.

The area where termite attack is high, the crop field and surroundings must be cleaned by removing all plant debris to maintain crop protection from termite attack. Pre‐planting tillage in the field, cleaning and cultivation of field borders destroys the termite reservoirs/mounds, reduces foraging activities of termites and improves plant health (Mahapatro & Chatterjee, 2018). Sowing of crops with high seed rate is essential to practice so that the removal of attacked and damaged plants from the field does not cause major economic losses (Mahapatro & Chatterjee, 2018). However, the high population of plant seedlings in the field may result in competition among plants for food, space, light and water. Additionally, this practice leads to an increase in seed cost. The regular intercultural practice through tillage in the field also minimizes termite damage by destroying termite galleries and mounds. Orchards must not be grown in sandy or sandy loam soil as termites preferred these types of soil.

Termites mostly attack weak plants, such as diseased or insect‐pest infected, mechanically injured or stressed plants (Diouf & Rouland‐Lefevre, 2018). The proper and balanced use of fertilizer, well‐decomposed farmyard manure, frequent irrigation and use of recommended agricultural practices increases plant vigor, which ultimately reduces termite attacks (Negassa & Sileshi, 2018). Paul *et al*. (2018) described that timely optimum use of nitrogen, phosphorous and potassium reduces termite attack in field crops. However, the misuse or incorrect application of commercially available fertilizers may result in salt accumulation, runoff, leaching, plant damage and soil pollution. Contrary to this, organic fertilizer or well‐decomposed manure not only enhances plant growth and reduces termite attack but also improves the soil structure and pH. However, it is very challenging to apply enough well‐decomposed farmyard manure as required to crops because of its low availability. Furthermore, it requires more labor during the application in the field. Negassa and Sileshi (2018) described that the integrated use of manure and fertilizer improves soil fertility, crop yield, prevents soil degradation and reduces the invasion of termites. They also concluded that the higher dose of fertilizer without organic input increased termite damage. Sane *et al*. (2016) proposed that water scarcity increases the mortality of the seedlings through its susceptibility to termite attack. However, the flooding or overuse of water for irrigation reduces plant growth and yield through waterlogging, nutrient leaching and runoff, increases root diseases and weed pressure and reduces air exchange between soil and atmosphere. Removal of weeds from the field resulted in a reduction of termite attack by increasing the plant vigor as weeds compete with crops for food, light, water and space which may leave the plant susceptible to termites.

Mulching with dead plant litter or green plant biomass in the field is a good strategy to keep crops less exposed to pest such as termites that are attracted to and feed on an alternate food source (Nyagumbo *et al*., 2015). Additionally, it reduces weed germination, increases soil fertility and improves soil water‐holding capacity. Mulching with *Cassia siamea* and *Azadirachta indica* suggests a very effective approach (Verma *et al*., 2009). Nyagumbo *et al*. (2015) described that mulching of maize residues in maize crop (*Zea mays*) reduces crop lodging due to termite attack as the termite preferentially fed on the maize residues. But there may be a possibility of an increase in the population of the termite due to excess availability of food. The use of plant material against termites is also an effective strategy to keep crops protected. The application of chopped plant leaf mixture (such as *Azadirachta indica*, *Tephrosia vogelii*, *Euphorbia tirucalli*, *Aloe graminicola*, *Melia azedarach*, *Lippie javanica*) and fruits (such as *Swartzia madagascariensis*) are commonly used practices worldwide to reduce the occurrence of termites in the field (Verma *et al*., 2009). Timely harvesting of the crops may also be a very effective and efficient practice to minimize termite damage.

Crop rotation and intercropping approaches are used to improve soil fertility, plant growth, natural enemy fauna and breakdown of the life cycle of termites by growing non‐preferred crops. The monoculture cropping system not only reduces soil fertility and structure but also reduces plant vigor and the ultimate increase in termite damage. Mahapatro and Chatterjee (2018) reported that push‐pull is a very effective technology for the manipulation in distribution and abundance of pests and beneficial insects in the field. According to them, after maize crop harvesting, sowing of wheat three rows in between the maize stubbles (0.75 m row spacing) may attract termites to feed on stubbles rather wheat crop, this practice not only reduced the termite damage on the wheat crop but also increased the soil fertility by decomposition of stubbles. The intercropping of maize with soybean or groundnut increases predatory ants and reduces the termite population. Girma *et al*. (2009) investigated the effectiveness of intercropping, mulching and their integration on termite infestation in maize crops. They used soybean as an intercropper and a mixture of neem seed powder along with maize stover as a mulch. Their results revealed that intercropping, mulching and combined application of intercropping + mulching not only reduced termite attacks but also enhanced maize crop yield. No doubt, crop rotation and intercropping are very good practices, but these are not effective in small‐scale areas. There should be some regional planting policies at the government level. Despite that, much more research is required in the area of intercropping, specific rotation and seasonal production breaks for better management of termites.

## Biological control measures

Biological control strategies included the use of predators, parasitoids/parasites or pathogens against termites to keep the population below a certain level.

### Predators

Some vertebrate and invertebrate predators kill termites generally on swarming reproductive alates and foraging workers. The invertebrate predators are ants, wasps, spiders, mantids, beetles, crickets, flies, dragonflies, cockroaches, centipedes, scorpions and so on, whereas the vertebrate predators are birds, mammals, amphibians, reptiles and so on. Among all predators, ants are the worst enemies of termites. Farmers in many regions of the world use sugar and leftover meat of animals as bait to increase the population of predatory ants in termite‐affected fields. Some species of ants such as *Iridomyrmex purpureus* (Oberst *et al*., 2017), *Plagiolepis pallescense*, *Polyrhachis lacteipenni*, *Pheidole teneriffana*, *Crematogaster antaris*, *Monomorium destructor* (Latifian *et al*., 2018), *Myrmicaria cumenoides*, *Pheidole megacephala*, *Leptogenys processionalis*, *Camponotus sericeus*, *Anoplolepis longipes*, and *Oecophulla smaragdina* (Paul *et al*., 2018), are the predatory ants of termites. Reduviids are the general predators of many arthropods and show preference to feed on termites. Gordon and Weirauch (2016) reported that many species of *Salyavatinae* (Hemiptera: Reduviidae) are the specialist predators of termites. The spiders, like *Ammoxenus amphalodes* are very specialized enemies of termites (Petráková *et al*., 2015).

### Pathogens

Microbial control is the exploitation of microorganisms (such as bacteria, fungi, nematodes and viruses) and their byproducts against insects. The biological control of termites with microbes gained very much attention during the past decades. Many researchers evaluate the effectiveness of entomopathogenic bacteria, fungi and nematodes against termites (Table 2). For example, the biological control fungus (*Metarhizium anisopliae*) has very successful results against termites (Balachander *et al*., 2013; Abonyo *et al*., 2016) and can be used as a potential biopesticide against termites. Some bacterial species such as *Bacillus* spp. provided good results against termites (Kalha *et al*., 2014). Similarly, some species of entomopathogenic nematode (*S. riobrave*, *S. carpocapsae*, *S. feltiae* and *H. bacteriophora*) infect subterranean termites (Yu *et al*., 2010). Minimal work has been done for the use of viruses as biological control agents (Chouvenc *et al*., 2011; Zhang & Mo, 2014). However, nuclear polyhedrosis virus (NPV) can be used as an important microbial agent against termites (Zhang & Mo, 2014).

**Table 2:** Potential entomopathogenic microorganisms against termites

Nematodes	References	Bacteria	References	Fungi	References
*Acrobeloides amurensis*	Carta *et al*., 2010	*Aeromonas caviae*	Devi, 2012	*Aspergillus* sp.	Pandey *et al*., 2012
*Heterorhabditis amazoniensis*	Kanga *et al*., 2012	*Alcalibenes latus*	Devi, 2012	*Beauveria bassiana*	Sileshi *et al*., 2013; Rana & Dinesh, 2014; Azmi *et al*., 2016
*Heterorhabditis bacteriophora*	Zamoum *et al*., 2011	*Bacillus cereus*	Khucharoenphaisan *et al*., 2012	*Beauveria brongniartii*	Yanagawa *et al*., 2012
*Heterorhabditis baujardi*	El‐Bassiouny & El‐Rahman, 2011	*Bacillus licheniformis*	Natsir & Dali, 2014	*Isaria fumosorosea*	Wright & Lax, 2013; Jessica *et al*., 2019
*Heterorhabditis indica*	Baimey *et al*., 2015	*Bacillus megaterium*	Omoya & Kelly, 2014	*Metarhizium anisopliae*	Ravindran *et al*., 2015; Yii *et al*., 2015; Azmi *et al*., 2016
*Heterorhabditis sonorensis*	Zadji *et al*., 2014a; Baimey *et al*., 2015	*Bacillus subtilis*	Omoya & Kelly, 2014	*Paecilomyces* sp.	Azmi *et al*., 2016
*Panagrolaimus spondyli*	Carta *et al*., 2010	*Bacillus thuiringiensis* subsp. *israelensis*	Singha *et al*., 2010; Wang & Henderson, 2013	*Paecilomyces fumosoroseus*	Rana & Dinesh, 2014; Jessica *et al*., 2019
*Poikilolaimus carsiops*	Kanzaki *et al*., 2011	*Bacillus thuiringiensis* subsp. *thuiringiensis*	Wang & Henderson, 2013	*Paecilomyces lilacinus*	Sharma *et al*., 2013
*Poikilolaimus scheffrahni*	Kanzaki *et al*., 2014	*Burkholderia cepacia*	Devi, 2012		
*Pelodera scrofulata*	Tahseen *et al*., 2014	*Candida utilis*	Khucharoenphaisan *et al*., 2012		
*Pelodera termitis*	Carta *et al*., 2010	*Citrobacter freundii*	Omoya & Kelly, 2014		
*Pseudaphelenchus sui*	Kanzaki *et al*., 2014	*Entrobacter cloacae*	Zhang *et al*., 2010		
*Pseudaphelenchus vindai*	Kanzaki *et al*., 2011	*Escherichia coli*	Khucharoenphaisan *et al*., 2012		
*Steinernema abbasi*	Abd‐Elbary *et al*., 2012	*Photorhabdus luminescens*	Shahina *et al*., 2011		
*Steinernema arenarium*	Abd‐Elbary *et al*., 2012	*Pseudomonas aeruginosa*	Khucharoenphaisan *et al*., 2012		
*Steinernema* sp.	Baimey *et al*., 2015	*Rhizobium leguminosarum*	Devi, 2012		
*Steinernema feltiae*	Zamoum *et al*., 2011	*Staphylococcus aureus*	Khucharoenphaisan *et al*., 2012		
*Steinernema glaseri*	Murugan & Vasugi, 2011				
*Steinernema karii*	Wagutu, 2017				
*Steinernema nyetense*	Kanga *et al*., 2012				
*Steinernema pakistanense*	Shahina & Tabassum, 2010				
*Steinernema riobrave*	Yu *et al*., 2010				

### Bacteria

Among microorganisms, bacterial pathogens are the first candidate evaluated as a biological control agent against many agricultural pests. Most pathogenic bacteria belong to the families Bacillaceae, Enterobacteriaceae, Pseudomonadaceae and Streptococcaceae (Kalha *et al*., 2014). However, considerable work has been done on only species of Bacillaceae, specifically *Bacillus* spp. The *B. thuringiensis* and *B. thuringiensis* subsp. *israelensis* are very effective pathogens against termites (*Microtermes obesi* and *Microcerotermes beesoni*) and can cause more than 80% mortality under laboratory conditions (Singha *et al*., 2010). Omoya and Kelly (2014) reported that *B. subtilis* is a very effective pathogen of some species of termite. Natsir and Dali (2014) suggested that *B. licheniformis* shows good pathogenicity against termites when used as feeding (baits) and spraying. Omoya and Kelly (2014) also suggested *S. marcescens* for the management of termites. Some species of rhizobacteria produce hydrogen cyanide (HCN) that can be used for management of subterranean termites (Devi, 2012; Yadav *et al*., 2016). Devi (2012) found satisfactory results of three HCN‐producing rhizobacteria species (i.e., *Aeromonas caviae*, *Alcaligenes latus* and *Rhizobium radiobacter*) against *Odontotermes obesus* under *in vitro* conditions. Devi and Kothamasi (2009) reported that *Pseudomonas fluorescens* causes mortality in termites by blocking the respiratory system and the production of HCN.

### Fungi

Entomopathogenic fungi have a very significant role in biological control of agricultural insect pests. These cosmopolitan organisms are isolated from soil and can cause mortality in all development stages of their hosts. More than 700 species of fungi have been described as pathogenic, and around 90 genera have been evaluated as entomopathogenic against insect pests of crops (Hemasree, 2013). Some well‐known genera, that is, *Antennopsis*, *Beauveria*, *Coreomyceptosis*, *Laboulbenia*, *Leboulbeniopsis*, *Metarhizium*, *Metirolella*, *Neotermus*, and *Termitaria* are pathogenic to termites. However, the pathogenicity relies on their asexual spores or conidia ability to disperse, transmit, adhere, germinate, penetrate the host cuticle for fungal growth and infection, and compete with other fast‐growing opponents and disease outbreak in the termite colony (Yii *et al*., 2015). The spores may be applied as a powder or through baits, either on termites or on nests.

Extensive effort has been made worldwide to evaluate *Beauveria bassiana* and *Metarhizium anisopliae* against termites. The fungi *M. anisopliae* effectively control different termite species such as *Coptotermes formosanus*, *Odontotermes* sp. and *Reticulitermes* sp. (Dong *et al*., 2009). In 2009, Balachander and coworkers reported that the isolates of *M. anisopliae* are pathogenic against termites (*Odontotermes* sp.) (Balachander *et al*., 2009). Furthermore, Balachander and coworkers used five isolates of the *M. anisopliae* spores with the attractants such as sugarcane bagasse, sawdust and cardboard powder against *Odontotermes obesus* and found >95% mortality in workers and >75% in soldiers with IWST‐Ma13 isolate after 13 days (Balachander *et al*., 2013). Ravindran *et al*. (2015) tested four isolates of *M. anisopliae* against *Coptotermes formosanus* and observed mortality in termites after 4 days. Hussain and coworkers experimented to find the efficacy of ARSEF6911 isolate of *M. anisopliae* in laboratory and field conditions against two species of termite, *M. obesi* and *O. obesus* (Hussain *et al*., 2011). They observed a significant reduction in the termite population in the laboratory as well as in the field. Samsuddin *et al*. (2016) evaluated ten isolates of *M. anisopliae* against *Coptotermes curvignathus* and found more than 95% mortality in termites. They also observed the fungal progression rate and conidia sporulation. The highest rate of mycelia formation was more than 85%, and conidia sporulation was 80% recorded against TFFH3 and PKLG isolates, respectively. Based on the performance of these isolates, they recommended the use of PR1 isolate as a potent biopesticide against termites. Yii and coworkers used the fipronil termiticide with *M. anisopliae* spores in combination and separately, against *Coptotermes curvignathus* (Yii *et al*., 2015). They concluded that the mixture of conidia and sublethal fipronil could cause more than 99% mortality in *C. curvignathus*. Sileshi *et al*. (2013) used four isolates from *M. anisopliae* and *B. bassiana* against *Macrotermes* sp. and observed 100% mortality of termites by all isolates after 7 days. They suggested that *M. anisopliae* and *B. bassiana* can be used as environmentally safe biopesticides. Rana and Kachhawa tested the spores of three fungi species (*M. anisopliae*, *B. bassiana* and *Paecilomyces fumosoroseus*) with farmyard manure (FYM) separately, for *in vivo* suppression of termites in a maize field. According to them, the soil applied with FYM + fungi spores at the time of sowing of the maize crop, provided better germination, yield and plant survival as compared to control (Rana & Dinesh, 2014). Pandey *et al*. (2013) and Wright and La (2013) described that the extensive exposure of *Aspergillus* sp. and *Isaria fumosorosa* caused rapid mortality of termites.

Although many researchers have confirmed that entomopathogenic fungi are very successful biological control microorganisms for the management of crop termites, few studies proved their failure for termite management. As we know, termites are highly sensitive to environmental abiotic factors. They have very well‐developed multidefense strategies against fungal pathogens (Liu *et al*., 2019). They prevent the entrance of pathogens into the colonies by avoiding fungus‐infected areas and fungus‐infected individuals, releasing antifungal secretions, and using symbiotic microorganism for nest materials (Liu *et al*., 2019). In case of pathogens entering into the colony, a well‐developed olfactory sense plays a key role in identifying and removing lethal conidial spores (Yanagawa *et al*., 2009). Furthermore, they eliminate pathogens quickly by grooming (Poulsen, 2015), antifungal secretions, aggressive behavior, cannibalism, burial (Liu *et al*., 2019) and social immunization (Liu *et al*., 2015; Cremer *et al*., 2018). Further, detoxification/antioxidation also play an important role in improving social immunity (Cremer *et al*., 2018; Liu *et al*., 2019). Hussain and Mingyi (2013) found the failure of fungus conidia for termite management under field conditions.

Keeping in mind all achievements and failure of fungi, we concluded that entomopathogenic fungi could be used as an effective and environmentally safe IPM strategy for the management of termites. As we discussed before in this review paper, the effectiveness of the fungi depends on their spore dispersion, transmission, germination, penetration into the host and environmental conditions (Yii *et al*., 2015). So, through improving these factors, termite management using fungi can be an effective method in an IPM program.

### Nematodes

Entomopathogenic nematodes (EPNs) are very important parasites and have the potential for the control of many agricultural insects in soil habitat. These pathogens are used as biological control agents to kill Macrotermitinae (Lin *et al*., 2015). However, they are very effective against termites under laboratory conditions, and no study has proved their usefulness under field conditions. The families of EPNs such as Steinernematidae and Heterorhabditidae are the important obligate insect parasites and can be used as efficient pathogens against termites. Yu *et al*. (2010) used three strains of *S. riobrave* against subterranean termite species *C. formosanus*, *Heterotermes aureus* and *R. flavipes*. Their results revealed that *S. riobrave* is more effective against *Heterotermes aureus* after 4 days of treatment. Zadji *et al*. (2014b) used four isolates of EPNs (*H. indica* Ayogbel, *H. sonorensis* Azohoue, *H. sonorensis* Ze3 and *Steinernema* sp. Bembereke) against *Macrotermes bellicosus* and *Trinervitermes occidentalis* species of termite. They described that both species showed susceptibility to all EPN isolates. Shahina and Tabassum (2010) experimented with laboratory conditions to reveal the efficacy of *S. pakistanense* against subterranean termite *Macrotermes*. According to their results, the termite was more susceptible to EPN strains. Murugan and Vasugi (2011) used a mixture of EPNs and neem seed kernel extract against *R. flavipes* and gained more effective results. Rathour and coworkers evaluated the EPN‐based biopesticide (Pusa Nemagel) against subterranean termites of wheat and pearl millet crops. They used this biopesticide at the time of sowing with soil application and observed more than 75% mortality as compared to control (Rathour *et al*., 2014).

Although many scientists under laboratory conditions evaluated EPNs, their compatibilities with pesticides and many other features make them applicable as a biological control approach. EPNs can be mass‐produced and formulated as microbial biopesticides for commercial production.

### Virus

Although some viruses have great potential to be a good microbial control pathogen against agricultural insects, they have gained very little attention worldwide (Chouvenc *et al*., 2011). Very few studies have been done to investigate the efficacy of viruses against termites under laboratory conditions. NPV is thought to be an important microbial agent against termites. However, its effectiveness against termites under field conditions is yet to be explored. Zhang and Mo (2014) determined the toxic effect of *Autographa californica* NPV (AcNPV) on workers of *Coptotermes formosanus* Shiraki. They treated filter paper or *Pinus massoniana* wood dust with different concentrations of NPV and fed these to termites. Their results revealed that AcNPV has a strong infection capacity against termites. Further, they suggested that *P. massoniana* wood dust treated with NPV is a very effective practice for termite control.

Although microbial control of termites via entomopathogenic bacteria, fungi and nematodes has very successful results and can be used as potential biopesticides against termites, there are some limitations to the application of these microorganisms. For example, they become inactive during unfavorable conditions. These microbes also caused soil and water pollution through leaching. Furthermore, the usage of these entomopathogens as biopesticides also have some complications with environmental factors (such as temperature, humidity, sunlight, rainfall, etc.) reducing their pesticidal potential through weakened persistence. Likewise, we cannot store these microbial pesticides for a long time as these biocontrol agents require very sophisticated storage systems which ultimately increases the application costs.

## Botanicals

Botanicals are phytobased products or plant‐derived pesticides that are considered as the most potential substitutes of highly harmful synthetic pesticides, derivative of plant roots, stems, leaves, flowers, fruits, seeds and wood. More than 2000 species of plants belonging to 60 families have pesticidal activities, many of them are used as insect growth regulators, ecdysones, behavior modulators, feeding deterrents, repellents, attractants and so on. (Verma *et al*., 2018). Many researchers have investigated the effects of plant‐derived materials against termites under laboratory and field conditions. Alshehry and coworkers tested hexane leaf extract of four plant species *Heliotropium bacciferum*, *Lantana camara*, *Rhazya stricta* and *Ruta chalepensis* against subterranean termite *Psammotermes hybostoma* and found all plants are promising against termites (Alshehry *et al*., 2014). Ibrahim and Demisse (2013) used 11 plants against termites of hot pepper and confirmed that *Maesa lanceolanta* and *Azadirachta indica* were very effective. Seo *et al*. (2014) experimented to estimate the fumigant toxicity of four plants including *Chamaemelum nobile*, *Eriocephalus punctulatus*, *Ormenis multicaulis* and *Santolina chamaecyparissus* against the termite *Reticulitermes speratus*. All plants provided strong fumigant toxicity against termites, but *Chamaemelum nobile* proved to be the best after 2 days of treatment. Addisu *et al*. (2013) used the leaf extract of plants *Azadiractin indica* and *Jatropha curcas* against *Macrotermes* spp. and found that the extract has potential against termites. Verma *et al*. (2013) investigated the effect of methanolic extract of *Jatropha curcas* against termites with satisfactory results. The reduction in the termite attack on *Triplochiton scleroxylon* and *Vitex doniana* wood was recorded, when treated with stem bark and leaf extracts of *Lawsonia inermis* (Adedeji *et al*., 2017). Bajya and coworkers conducted a laboratory experiment to investigate the repellent activities of *Crotalaria burhia* root extract and *Anacardium occidentale* leaf extract against *O. obesus*. They reported that *C. burhia* repelled more than 70% of *O. obesus* whereas *A. occidentale* repelled a maximum 60% of *O. obesus* (Bajya *et al*., 2015). The seed extracts of plant species *Chenopodium ambrosioides*, *Maesa lanceolate* and *Vernonia hymenolepis* significantly reduced *Macrotermes* sp. attack (Addisu *et al*., 2013). The plant species *Andrographis lineata*, *Aristolochia bracteolate*, *Datura metel* and *Eclipta prostrata* have anti‐termitic activities (Elango *et al*., 2012). Hu *et al*. (2015) experimented to investigate the effectiveness of *Camellia oleifera* against *Reticulitermes flavipes* and found satisfactory results. *Lantana camara* leaf extracts are antifeedant, repellent and toxic against *Reticulitermes flavipes* (Yuan & Hu, 2012). Qureshi and coworkers used a different concentration of *Melia azedarach* in water and methanol against *M. obesi* and *O. obesus* and found effective results against *O. obesus* (Qureshi *et al*., 2015). Ahmed *et al*. (2016) used three concentrations, that is, 5%, 10% and 20% from a different solutions including garlic, neem and tobacco against *Heterotermis indicola* under laboratory conditions, and better results were recorded for all solutions, even though high mortality by garlic and tobacco was reported. Liu *et al*. (2015) estimated the capability of leaf hexane extract of *Aristolochia bracteolate*, ethyl acetate extract of *A. paniculata*, *Datura metel* and *Euphorbia prostrata*, or methanol extract of *Acacia lineata* and *D. metel* against termites. They reported that all treatments cause promising mortality after 1 day of application.

Various plant chemicals and secondary metabolites, for example alkaloids, steroids, essential oils, terpenoids, flavonoids, resins and so on are used for effective control of termites. Xie *et al*. (2014) extracted the monoterpenes from aromatic plants and investigated their potential against termites with satisfactory results. Himmi and coworkers tested neem oil against *Coptotermes gestroi* and found that azadirachtin fraction with 91% purity provides maximum mortality against termites. Himmi *et al*. (2013) investigated the fumigant toxicity of essential oils from four plants, that is, *Chamaemelum nobile*, *Eriocephalus punctulatus*, *Ormenis multicaulis* and *Santolina chamaecyparissus* against the Japanese termite *Reticulitermes speratus* and they found strong results after 2 days of treatment (Seo *et al*., 2014). Verma *et al*. (2016) reported that the plant essential oils of marigold and sweet orange have strong repellency. Lima *et al*. (2013) used essential oils from seven plant species *Corymbia citriodors*, *Croton sonderianus*, *Cymbopogon martini*, *Lippie alba*, *L. gracilis*, *L. sidoides* and *Pogostemon cablin* against *Nasutitermes corniger* and observed that all essential oils were effective against workers of termites. The seed oil of *Jatropha* plant caused 100% mortality against *Odentotermes obesus* after 48 h of treatment (Ede & Demissie, 2013). Pandey and coworkers used essential oils of seven plants *Cymbopogon citratus*, *Eucalyptus globulus*, *Syzygium aromaticum*, *Origanum vulgare*, *Rosmarinus offcinalis*, *Cinnamomum verum* and *Thymus vulgaris* against *Odentotermes assamensis*. They investigated that phenolic compounds exhibited effective results compared with other compounds. They also described that acetate, alcohol and aldehyde provided satisfactory results. Zhou *et al*. (2008a) tested three novel carbohydrate‐based enzyme inhibiters, that is, cellobioimidazole (CBI), fluoromethyl cellobiose (FMCB) and fluoromethyl glucose (FMG) against *R. flavipes*. Their results revealed that FMCB and CBI have potential as termite control agents. Some plants are resistant to many subterranean species of termites (Pandey *et al*., 2012). For example, two plant species *Khaya ivorensis* and *K. senegalen* exhibited natural resistance against termites (França *et al*., 2016). Great work has been done on phyto‐based material against insect pests during the past decades but the compatible combination of these chemicals is yet to be explored. Furthermore, widespread work is required for the development of clear concentration and application rates of these plant extracts. Besides, the commercially available products are much too expensive and protect for only a short period. No doubt, we cannot compare plant extracts with chemical pesticides. However, they are less injurious to the environment and can be used as one of the best strategies in IPM of termites.

## Chemical control measures

Even though chemicals are hazardous to the environment, farmers all over the world use them extensively for the management of agricultural pests. Management of termites with termiticides is a difficult task as termites are eusocial insects and live in mounds or many inches below the soil surface to keep themselves protected from outside threats. However, some termiticides such as imidacloprid, chlorpyrifos, fipronil, spinosad, chlorfenapyre, bifenthrin, cypermethrin, permethrin, disodium octaborate tetrahydrate, calcium arsenate, lindane, endosulfan and chlorantraniliprole have been used worldwide for the management of termites. Generally, farmers use these termiticides during irrigation of the crops. However, injection of termiticides is also used for the control of termites in field crops, forests and buildings. Some fumigants such as carbon dioxide, methyl bromide, sulfur fluoride and phosphine are also used through fumigation methods for the control of termites attacking dry woods and stored grain products. Iqbal and Saeed (2013) investigated the efficacy of six insecticides, imidacloprid, indoxacarb, fipronil, spinosad, thiamethoxam and chlorfenapyre against *Microtermes mycophagus*. They reported that all insecticides provided satisfactory results, but chlorfenapyre and spinosade were the most potent among all. Ahmed *et al*. (2015) tested insecticides against *Psammotermes hypostoma* and found chlorpyrifos, acetamiprid and thiamethoxam the most effective insecticides for the control of termites. Bhagawati *et al*. (2014) used clothianidian and a mixture of acephate + imidacloprid to treat sugarcane setts under field conditions. They described that clothianidin efficiently reduced termite infestation, statistically the same as with the combined application of acephate + imidacloprid. Manzoor *et al*. (2014) conducted a study under laboratory conditions to evaluate the toxicity and repellency of imidacloprid against *Microtermes obesi*. They reported that imidacloprid is a non‐repellent insecticide, and caused more than 90% mortality. They also investigated the tunneling behavior of termites, and their results revealed that high concentration of imidacloprid caused a reduction in tunnel numbers and cumulative tunnel distance. Saljoqi *et al*. (2014) tested three insecticides fipronil, spinosad and lufenuron against subterranean termites *Heterotermes indicola*. They described that all insecticides were effective against termites, but fipronil and spinosad proved to be the best. Chen and coworkers assessed the toxicity of ten insecticides, chlorpyrifos, phoxim, carbofuran, propoxur, bifenthrin, cypermethrin, imidacloprid, fipronil, abamectin and ivermectin against *Reticulitermes speratus* under laboratory conditions. They documented that all insecticides are effective against termites, but abamectin, ivermectin, fipronil and imidacloprid showed strong toxicity (Chen *et al*., 2015). Wang *et al*. (2014) conducted a laboratory study to reveal the sublethal effects of lufenuron insecticide on *Coptotermes formosanus* physiology and behavior. Their results showed that all concentrations significantly reduced survivorship, running speed, food consumption and tunneling. Furthermore, they also found that application of lufenuron inhibited the carcass‐burying and particle transport behavior of the termites. Grimball *et al*. (2017) described that cyclohexylamine is a novel termiticide, and its hydrogen phosphate salts maintain its toxicity. Bhatta *et al*. (2016) tested the toxicity and repellency of spinosad and spinetoram against *Coptotermes formosanus* under laboratory conditions and reported that both insecticides exhibited toxicity and non‐repellency against termites. Huang *et al*. (2006) evaluated fipronil bait consisting of straw pulp and white sugar, against *O. formosanus* under field conditions and found satisfactory results. Huang and Lei (2005) conducted an experiment to investigate the possibility of transfer of fipronil from exposed workers of *O. formosanus* to unexposed nestmates. To this purpose, they used a simple donor‐recipient model. Their results revealed that when 15 to 20 donors treated with 5 ppm fipronil and exposed for 6 h duration provided significant mortality of recipients. Li *et al*. (2010) tested three different baits, that is, sulfluramid baits, hexaflumuron baits and fipronil baits against subterranean termite, *Reticulitermes chinensis* in rural houses of China with satisfactory results.

No doubt, termites can be controlled effectively through chemical approaches, but we cannot forget their injurious effects on humans, animals and birds. Further, the indiscriminate use of pesticides causes pest resistance and environmental pollution.

## RNA interference

RNA interference (RNAi) is a conserved biological process and manipulation of the post‐transcriptional gene silencing triggered by double‐standard RNA (dsRNA) (Gu & Knipple, 2013). It is a powerful and viable technology of functional genomics that has recently been used to control highly targeted agricultural insect pests (Burand & Hunter, 2013). The ability to target genes in a species‐specific manner (Gu & Knipple, 2013) makes this approach a potential tool for termite control. However, very few studies have been conducted previously to explore\RNAi for termite management. The first RNAi study in termites was conducted by Zhou *et al*. (2006). They injected short interfering RNAs (siRNAs) into *R. flavipes*. Later, Zhou *et al*. (2008b) used the dsRNA feeding approach in *R. flavipes* to target two termite genes. These genes included: (1) endoglucanase gene, which encodes cellulase digestive enzyme; and (2) hexamerin gene, which encodes a caste regulatory hexamerin storage protein. Wu and Li (2018) injected dsRNA directed against *β*‐glucosidase genes (*CfBG‐Ia* and *CfBG‐Ib*) in *C. formosanus* workers and found a significant decrease in gene expression but minimal lethal impact. Hamilton and Bulmer (2012) described that Termicin and GNBP2 proteins have a direct role in external antifungal defense strategy. They ingested dsRNA targeted against Termicin and GNBP2 in Reticulitermes subterranean termite. Their results indicated that there was a significate decrease in gene expression and increase in mortality among termites exposed to *M. anisopliae*.

Although researchers have successfully been using RNAi to characterize genes in termites through injection and feeding of dsRNA, still more innovative efforts are needed for its potential use as a target‐specific control strategy against termites. Recently, Wu *et al*. (2019) investigated the effect of RNAi on termites by targeting the five endoglucanase genes (*GfEG*s) in *C. formosanus* workers. They introduced dsRNA in termites through both injection and oral delivery. They found a significant increase in mortality, decrease in enzyme activity and weight. Further, they fed *dsCfEG* with flufenoxuron to workers and obtained more good results as compared to the *dsCfEG* or flufenoxuron only treatment.

## Conclusion

Termites are becoming a threat to the world farming community. Significant work has been done on various components of ITM, but further innovative efforts are still needed. Termite management is still a great challenge because they are always hidden in mounds or underneath the soil and it is difficult to reach their target places. Following the well‐known proverb “Prevention is better than cure” farmers should improve their cultural management practices. The integration of traditional practice with recently developed approaches can be an effective strategy for its management. No doubt, some termiticides have been proven effective against termites, but their use should be the last option in an ecological management approach as these are very injurious to humans and the environment. We discussed earlier in this review paper that entomo‐pathogens, botanicals, toxic and non‐toxic barriers, mulching, dequeening, clean cultivation, plant vigor improvement, intercropping, specific rotation and use of baits are not only effective against termites but also environment‐friendly as proven by various researchers all over the world. However, widespread dissemination of these termite IPM practices is much needed as farmers are unaware of these approaches. We can achieve this difficult task by encouraging and strengthening communication and coordination among researchers and extension officers.

## Disclosure

The authors declare they have no conflicts of interest.

## References

[ins12726-bib-0001] Abd‐Elbary, N. , Shamseldean, M. , Stock, S. and Abu‐Shady, N. (2012) Diversity of entomopathogenic nematode species (Heterorhabditidae and Steinernematidae) in Egypt. Egyptian Journal of Agronematology, 11, 333–353.

[ins12726-bib-0002] Abonyo, E. , Maniania, N. , Warui, C.M. , Kokwaro, E. , Palmer, T. , Doak, D . *et al* (2016) Effects of entomopathogenic fungus *Metarhizium anisopliae* on non‐target ants associated with *Odontotermes* spp. (Isoptera: Termitidae) termite mounds in Kenya. International Journal of Tropical Insect Science, 36, 128–134.

[ins12726-bib-0003] Acda, M.N. (2013) Geographical distribution of subterranean termites (Isoptera) in economically important regions of Luzon, Philippines. The Philippine Agricultural Scientist, 96, 205–209.

[ins12726-bib-0004] Addisu, S. , Waktole, S. and Mohamed, D. (2013) Laboratory evaluation of entomopathogenic fungi *Metarhizium anisophilae* and *Beauveria bassiana* against termite, *Macrotermes* (Isoptera: Termitidae). Asian Journal of Plant Sciences, 12, 1–10.

[ins12726-bib-0005] Adedeji, G.A. , Ogunsanwo, O.Y. and Elufioye, T.O. (2017) Quantifications of phytochemicals and biocide actions of *Lawsonia inermis* Linn. Extracts against wood termites and fungi. International Biodeterioration & Biodegradation, 116, 155–162.

[ins12726-bib-0006] Ahmed, M.A.I. , Eraky, E.S.A. , Mohamed, M.F. and Soliman, A.A.S. (2015) Potential toxicity assessment of novel selected pesticides against sand termite, *Psammotermes hypostoma* (Desneux workers) (Isoptera: Rhinotermitidae) under field conditions in Egypt. Journal of Plant Protection Research, 55, 193–197.

[ins12726-bib-0007] Ahmed, N. , Huma, Z. , Rehman, S.U. , Ullah, M. and Ahmed, S. (2016) Effect of different plant extracts on termite species (*Heterotermis indicola*). Journal of Bioresource Management, 3, 2.

[ins12726-bib-0008] Alshehry, A.Z. , Zaitoun, A.A. and Abo‐Hassan, R.A. (2014) Insecticidal activities of some plant extracts against subterranean termites, *Psammotermes hybostoma* (Desneux)(Isoptera: Rhinotermitidae). International Journal of Agricultural Sciences, 4, 257–260.

[ins12726-bib-0009] Atsbha, G. and Hintsa, M. (2018) Evaluation of chemical, botanical and cultural management options of termite in Tanqua Abergelle district, Ethiopia. African Journal of Plant Science, 12, 98–104.

[ins12726-bib-0010] Azmi, W.A. , Sulaiman, Z.A. , Ishak, I. , Kin, P.K. , Lin, G.L.E. and bt Addis, S.N.K. (2016) Virulence evaluation of entomopathogenic fungi to subterranean termites, *Globitermes sulphureus* (Insecta: Isoptera). Malaysian Journal of Microbiology, 12, 492–497.

[ins12726-bib-0011] Baimey, H. , Zadji, L. , Afouda, L. , Moens, M. and Decraemer, W. (2015) Influence of pesticides, soil temperature and moisture on entomopathogenic nematodes from southern Benin and control of underground termite nest populations. Nematology, 17, 1057–1069.

[ins12726-bib-0012] Bajya, D. , Manoharan, T. , Sridharan, S. and Kuttalam, S. (2015) Repellent efficacy of *Crotalaria burhia* and *Anacardium occidentale* against *Odontotermes obesus* (Isoptera: Termitidae) under laboratory conditions. The Indian Journal of Agricultural Sciences, 85, 1234–1236.

[ins12726-bib-0013] Balachander, M. , Remadevi, O. and Sasidharan, T. (2013) Dissemination of *Metarhizium anisopliae* infection among the population of *Odontotermes obesus* (Isoptera: Termitidae) by augmenting the fungal conidia with attractants. Journal of Asia‐Pacific Entomology, 16, 199–208.

[ins12726-bib-0014] Balachander, M. , Remadevi, O. , Sasidharan, T. and Bai, N.S. (2009) Infectivity of *Metarhizium anisopliae* (Deuteromycotina: Hyphomycetes) isolates to the arboreal termite *Odontotermes* sp. (Isoptera: Termitidae). International Journal of Tropical Insect Science, 29, 202–207.

[ins12726-bib-0015] Bhagawati, S. , Bhattacharyya, B. , Mishra, H. and Gogoi, D. (2014) Chemical management of termites (*Odontotermes obesus*) in preserved setts of sugarcane (*Saccharum officinarum*). Journal of Entomology and Zoology Studies, 5, 856–859.

[ins12726-bib-0016] Bhatta, D. , Henderson, G. and Gautam, B. (2016) Toxicity and nonrepellency of spinosad and spinetoram on formosan subterranean termites (Isoptera: Rhinotermitidae). Journal of Economic Entomology, 109, 1341–1349. 27106223 10.1093/jee/tow079

[ins12726-bib-0017] Bonachela, J.A. , Pringle, R.M. , Sheffer, E. , Coverdale, T.C. , Guyton, J.A. , Caylor, K.K . *et al* (2015) Termite mounds can increase the robustness of dryland ecosystems to climatic change. Science, 347, 651–655. 25657247 10.1126/science.1261487

[ins12726-bib-0018] Brauman, A. , Majeed, M.Z. , Buatois, B. , Robert, A. , Pablo, A.L. and Miambi, E. (2015) Nitrous oxide (N_2_O) emissions by termites: does the feeding guild matter? PLoS ONE, 10, e0144340. 26658648 10.1371/journal.pone.0144340 PMC4675541

[ins12726-bib-0019] Brune, A. (2014) Symbiotic digestion of lignocellulose in termite guts. Nature Reviews Microbiology, 12, 168–180. 24487819 10.1038/nrmicro3182

[ins12726-bib-0020] Burand, J.P. and Hunter, W.B. (2013) RNAi: future in insect management. Journal of Invertebrate Pathology, 112, S68–S74. 22841639 10.1016/j.jip.2012.07.012

[ins12726-bib-0021] Cao, R. and Su, N.Y. (2015) Temperature preferences of four subterranean termite species (Isoptera: Rhinotermitidae) and temperature‐dependent survivorship and wood‐consumption rate. Annals of the Entomological Society of America, 109, 64–71.

[ins12726-bib-0022] Carta, L. , Handoo, Z. , Lebedeva, N. , Raina, A. , Zhuginisov, T. and Khamraev, A.S. (2010) *Pelodera termitis* sp. n. and two other rhabditid nematode species associated with the Turkestan termite *Anacanthotermes turkestanicus* from Uzbekistan. International Journal of Nematology, 20, 125–134.

[ins12726-bib-0023] Chand, R.R. , Jokhan, A.D. , Charan, H. , Raj, K. and Singh, P. (2018) Threats posed by Asian subterranean termites in the Fiji Islands and their potential controls: a review. New Zealand Plant Protection, 71, 129–139.

[ins12726-bib-0024] Chen, Z. , Qu, Y.Y. , Xiao, D. , Song, L.F. , Zhang, S.H. , Gao, X.W . *et al* (2015) Lethal and social‐mediated effects of ten insecticides on the subterranean termite *Reticulitermes speratus* . Journal of Pest Science, 88, 741–751.

[ins12726-bib-0025] Chouvenc, T. , Su, N.Y. and Grace, J.K. (2011) Fifty years of attempted biological control of termites—Analysis of a failure. Biological Control, 59, 69–82.

[ins12726-bib-0026] Cremer, S. , Pull, C.D. and Fuerst, M.A. (2018) Social immunity: emergence and evolution of colony‐level disease protection. Annual Review of Entomology, 63, 105–123. 10.1146/annurev-ento-020117-043110 28945976

[ins12726-bib-0027] Devi, K.K. (2012) Investigations on cyanide producing pseudomonad bacterial spieces and their potential for application against termite *Odontotermes obesus* *. University of Delhi*, 93p, http://hdl.handle.net/10603/13643.

[ins12726-bib-0028] Devi, K.K. and Kothamasi, D. (2009) *Pseudomonas fluorescens* CHA0 can kill subterranean termite *Odontotermes obesus* by inhibiting cytochrome c oxidase of the termite respiratory chain. FEMS Microbiology Letters, 300, 195–200. 19769587 10.1111/j.1574-6968.2009.01782.x

[ins12726-bib-0029] Diba, F. , Hadary, F. , Panjaitan, S.D. and Yoshimura, T. (2013) Electromagnetic waves as non‐destructive method to control subterranean termites *Coptotermes curvignathus* Holmgren and *Coptotermes formosanus* Shiraki. Procedia Environmental Sciences, 17, 150–159.

[ins12726-bib-0030] Diouf, M. and Rouland‐Lefevre, C. (2018) The fungus‐growing termites: Biology, damage on tropical crops and specific management Termites and Sustainable Management. Springer, Cham, pp. 1–35.

[ins12726-bib-0031] Dong, C. , Zhang, J. , Huang, H. , Chen, W. and Hu, Y. (2009) Pathogenicity of a new China variety of *Metarhizium anisopliae* (*M. anisopliae* var. *dcjhyium*) to subterranean termite *Odontotermes formosanus* . Microbiological Research, 164, 27–35. 17482440 10.1016/j.micres.2006.11.009

[ins12726-bib-0032] Ede, A.G. and Demissie, A.G. (2013) Comparative bio‐activity guided characterization of biocide from *Jatropha curcas* and *Ricinus communis* L. seeds oil. Journal of Pharmacognosy and Phytochemistry, 2, 176–181.

[ins12726-bib-0033] El‐Bassiouny, A. and El‐Rahman, R.A. (2011) Susceptibility of Egyptian subterranean termite to some entomopathogenic nematodes. Egyptian Journal of Agricultural Research, 89, 121–135.

[ins12726-bib-0034] Elango, G. , Rahuman, A.A. , Kamaraj, C. , Bagavan, A. , Zahir, A.A. , Santhoshkumar, T . *et al* (2012) Efficacy of medicinal plant extracts against Formosan subterranean termite, Coptotermes formosanus. Industrial Crops and Products, 36, 524–530.

[ins12726-bib-0035] Evans, T.A. , Dawes, T.Z. , Ward, P.R. and Lo, N. (2011) Ants and termites increase crop yield in a dry climate. Nature Communications, 2, 262. 10.1038/ncomms1257 PMC3072065 21448161

[ins12726-bib-0036] Forschler, B. (2011) Sustainable termite management using an integrated pest management approach. Urban Pest Management: An Environmental Perpective, 133–144.

[ins12726-bib-0037] Forschler, B.T. , Jones, S.C. , Kard, B. , Baumann, G.J. , Henderson, G. , Suiter, D . *et al* (2007) Still an ongoing process. Pest Control, 75, 88–95.

[ins12726-bib-0038] França, T.S.F.A. , França, F.J.N. , Arango, R.A. , Woodward, B.M. and Arantes, M.D.C. (2016) Natural resistance of plantation grown African mahogany (*Khaya ivorensis* and *Khaya senegalensis*) from Brazil to wood‐rot fungi and subterranean termites. International Biodeterioration & Biodegradation, 107, 88–91.

[ins12726-bib-0039] Girma, D. , Addis, T. and Tadele, T. (2009) Effect of mulching and intercropping on termite damage to maize at Bako, Western Ethiopia. Pest Manag J Ethiop, 13, 38–43.

[ins12726-bib-0040] Gordon, E.R. and Weirauch, C. (2016) Efficient capture of natural history data reveals prey conservatism of cryptic termite predators. Molecular Phylogenetics and Evolution, 94, 65–73. 26314664 10.1016/j.ympev.2015.08.015

[ins12726-bib-0041] Govorushko, S. (2019) Economic and ecological importance of termites: A global review. Entomological Science, 22, 21–35.

[ins12726-bib-0042] Grimball, B. , Veillon, L. , Calhoun, T. , Fronczek, F.R. , Arceneaux, E. and Laine, R.A. (2017) Cyclohexylamine inexplicably induces antennae loss in Formosan subterranean termites (*Coptotermes formosanus* Shiraki): cyclohexylamine hydrogen phosphate salts are novel termiticides. Pest Management Science, 73, 2039–2047. 28485048 10.1002/ps.4610

[ins12726-bib-0043] Gu, L. and Knipple, D.C. (2013) Recent advances in RNA interference research in insects: Implications for future insect pest management strategies. Crop Protection, 45, 36–40.

[ins12726-bib-0044] Hadi, Y. , Massijaya, M. and Arinana, A. (2016) Subterranean termite resistance of polystyrene‐treated wood from three tropical wood species. Insects, 7, 37. 10.3390/insects7030037 PMC5039550 27455331

[ins12726-bib-0045] Hamilton, C. and Bulmer, M.S. (2012) Molecular antifungal defenses in subterranean termites: RNA interference reveals in vivo roles of termicins and GNBPs against a naturally encountered pathogen. Developmental & Comparative Immunology, 36, 372–377. 21824492 10.1016/j.dci.2011.07.008

[ins12726-bib-0046] Hemasree, E. (2013) A critical review on the natural occurrence of entomopathogenic fungi in agricultural ecosystem. International Journal of Applied Biology and Pharmaceutical Technology, 4, 372–375.

[ins12726-bib-0047] Himmi, S.K. , Tarmadi, D. , Ismayati, M. and Yusuf, S. (2013) Bioefficacy performance of neem‐based formulation on wood protection and soil barrier against subterranean termite, *Coptotermes gestroi* Wasmann (Isoptera: Rhinotermitidae). Procedia Environmental Sciences, 17, 135–141.

[ins12726-bib-0048] Hu, J. , Chang, S. , Peng, K. , Hu, K. and Thévenon, M.F. (2015) Bio‐susceptibility of shells of *Camellia oleifera* Abel. fruits to fungi and termites. International Biodeterioration & Biodegradation, 104, 219–223.

[ins12726-bib-0049] Huang, F.S. , Zhu, S.M. , Ping, Z.M. , He, X.S. , Li, G.X. and Gao, D.R. (2000) Fauna Sinica: Insecta, *Vol*. *17: Isoptera*. Science Press, Beijing, 961 pp.

[ins12726-bib-0050] Huang, Q.Y. and Lei, C.L. (2005) Transfer of fipronil from exposed workers of the subterranean termite *Odontotermes formosanus* (Isoptera: Termitidae) to unexposed nestmates. Sociobiology, 46, 385–395.

[ins12726-bib-0051] Huang, Q.Y. , Lei, C.L. and Xue, D. (2006) Field evaluation of a fipronil bait against subterranean termite *Odontotermes formosanus* (Isoptera: Termitidae). Journal of Economic Entomology, 99, 455–461. 16686147 10.1603/0022-0493-99.2.455

[ins12726-bib-0052] Hussain, A. , Ahmed, S. and Shahid, M. (2011) Laboratory and field evaluation of *Metarhizium anisopliae* var. *anisopliae* for controlling subterranean termites. Neotropical Entomology, 40, 244–250. 21584407

[ins12726-bib-0053] Hussain, A. and Mingyi, T. (2013) Germination pattern and inoculum transfer of entomopathogenic fungi and their role in disease resistance among *Coptotermes formosanus* (Isoptera: Rhinotermitidae). International Journal of Agriculture and Biology, 15.

[ins12726-bib-0054] Ibrahim, A. and Demisse, G. (2013) Evaluation of some botanicals against termites’ damage on hot pepper at Bako, Western Ethiopia. International Journal of Agricultural Policy and Research, 1, 48–52.

[ins12726-bib-0055] Iqbal, N. and Saeed, S. (2013) Toxicity of six new chemical insecticides against the termite, *Microtermes mycophagus* D. (Isoptera: Termitidae: Macrotermitinae). Pakistan Journal of Zoology, 45, 709–713.

[ins12726-bib-0056] Jessica, J. , Peng, T. , Sajap, A. , Lee, S. and Syazwan, S. (2019) Evaluation of the virulence of entomopathogenic fungus, *Isaria fumosorosea* isolates against subterranean termites *Coptotermes* spp. (Isoptera: Rhinotermitidae). Journal of Forestry Research, 30, 213–218.

[ins12726-bib-0057] Kalha, C. , Singh, P. , Kang, S. , Hunjan, M. , Gupta, V. and Sharma, R. (2014) Entomopathogenic viruses and bacteria for insect‐pest control Integrated Pest Management (ed. Abrol D.P.). pp. 225–244. Elsevier, India.

[ins12726-bib-0058] Kanga, F.N. , Waeyenberge, L. , Hauser, S. and Moens, M. (2012) Distribution of entomopathogenic nematodes in southern Cameroon. Journal of Invertebrate Pathology, 109, 41–51. 21983478 10.1016/j.jip.2011.09.008

[ins12726-bib-0059] Kanzaki, N. , Center, B. , Giblin‐Davis, R. , Kosaka, H. , Lan, Y.C. and Li, H.F. (2011) *Poikilolaimus carsiops* n. sp. (Rhabditida: Rhabditidae) associated with *Neotermes koshunensis* (Kalotermitidae) in Kenting National Park, Taiwan. Nematology, 13, 155–164.

[ins12726-bib-0060] Kanzaki, N. , Li, H.F. , Lan, Y.C. and Giblin‐Davis, R.M. (2014) Description of two *Pseudaphelenchus* species (Tylenchomorpha: Aphelenchoididae) associated with Asian termites and proposal of Tylaphelenchinae n. subfam. Nematology, 16, 963–978.

[ins12726-bib-0061] Keefer, T.C. , Zollinger, D.G. and Gold, R.E. (2013) Evaluation of aggregate particles as a physical barrier to prevent subterranean termite incursion into structures. Southwestern Entomologist, 38, 447–465.

[ins12726-bib-0062] Khucharoenphaisan, K. , Sripairoj, N. and Sinma, K. (2012) Isolation and identification of actinomycetes from termite's gut against human pathogen. Asian Journal of Animal and Veterinary Advances, 7, 68–73.

[ins12726-bib-0063] Lagendijk, D. , Davies, A. , Eggleton, P. and Slotow, R. (2016) No evidence for an elephant–termite feedback loop in Sand Forest, South Africa. Biological Conservation, 203, 125–133.

[ins12726-bib-0064] Latifian, M. , Habibpour, B. and Kard, B. (2018) Predator ants of the date palm termite *Microcerotermes diversus* Silvestri and effects of ant morphometric characteristics on ant functional response. American Journal of Entomology, 2, 16–22.

[ins12726-bib-0065] Lenz, M. , Sunden‐Bylehn, A. , Thorne, B. , Lewis, V. and Haverty, M. (2003) Finding alternatives to persistent organic pollutants (POPs) for termite management. *UNEP/FAO/Global IPM Facility Expert Group on Termite Biology and Management, United Nations Environment Programme/Food and Agriculture Organization of the United Nations/Global Integrated Pest Management Facility. United Nations Environment Programme*.

[ins12726-bib-0066] Li, H. , Xu, Z. , Deng, T. , Chen, L. , Li, J. , Wei, J. *et al* (2010) Species of termites (Isoptera) attacking trees in China. Sociobiology, 56, 109–120.

[ins12726-bib-0067] Li, J. , Hu, Y. , Deng, T.F. , Guo, J.Q. , Gong, Y.G. and Mo, J.C. (2011) Laboratory and field evaluation of gravel sand as a physical barrier against *Coptotermes formosanus* and *Reticulitermes flaviceps* (Isoptera: Rhinotermitidae). Sociobiology, 57, 71–78.

[ins12726-bib-0068] Li, K. , Jiang, Y. , Wu, G.H. , Zhou, X.M. and Niu, C.Y. (2001) The general termites and damage in garden of Wuhan City. Journal of Huazhong Agricultural University, 20, 547–549.

[ins12726-bib-0069] Li, W. , Tong, Y. , Xiong, Q. and Huang, Q. (2010) Efficacy of three kinds of baits against the Subterranean termite *Reticulitermes chinensis* (Isoptera: Rhinotermitidae) in rural houses in China. Sociobiology, 56, 209–222.

[ins12726-bib-0070] Li, Y. , Dong, Z.Y. , Pan, D.Z. , Pan, C.H. and Chen, L.H. (2017) Effects of termites on soil pH and its application for termite control in Zhejiang Province, China. Sociobiology, 64, 317–326.

[ins12726-bib-0071] Li, Z. , Feng, X. , Liu, S.S. , You, M. and Furlong, M.J. (2016) Biology, ecology, and management of the diamondback moth in China. Annual Review of Entomology, 61, 277–296. 10.1146/annurev-ento-010715-023622 26667272

[ins12726-bib-0072] Lima, J.K. , Albuquerque, E.L. , Santos, A.C.C. , Oliveira, A.P. , Araújo, A.P.A. , Blank, A.F . *et al* (2013) Biotoxicity of some plant essential oils against the termite *Nasutitermes corniger* (Isoptera: Termitidae). Industrial Crops and Products, 47, 246–251.

[ins12726-bib-0073] Lin, Y. , Fang, D. and Wang, L. (2015) Termites and microbial biological control strategies Muhammad Qasim. South Asia Journal of Multidisciplinary Studies, 1, 33–62.

[ins12726-bib-0074] Liu, G. , Cornwell, W.K. , Cao, K. , Hu, Y. , Van Logtestijn, R.S. , Yang, S . *et al* (2015) Termites amplify the effects of wood traits on decomposition rates among multiple bamboo and dicot woody species. Journal of Ecology, 103, 1214–1223.

[ins12726-bib-0075] Liu, L. , Li, G.L. , Sun, P.D. , Lei, C.L. and Huang, Q.Y. (2015) Experimental verification and molecular basis of active immunization against fungal pathogens in termites. Scientific Reports, 5, 15106. 26458743 10.1038/srep15106 PMC4602225

[ins12726-bib-0076] Liu, L. , Zhao, X.Y. , Tang, Q.B. , Lei, C.L. and Huang, Q.Y. (2019) The mechanisms of social immunity against fungal infections in eusocial insects. Toxins, 11, 244. 10.3390/toxins11050244 PMC6563085 31035652

[ins12726-bib-0077] Maayiem, D. , Bernard, B.N. and Irunuoh, A.O. (2012) Indigenous knowledge of termite control: A case study of five farming communities in Gushegu District of Northern Ghana. Journal of Entomology and Nematology, 4, 58–64.

[ins12726-bib-0078] Mahapatro, G. and Chatterjee, D. (2018) Integrated termite management in the context of indoor and outdoor pest situation Termites and Sustainable Management. Springer, Cham, pp. 119–135.

[ins12726-bib-0079] Manzoor, F. , Saleem, S. and Abbas, M. (2014) Laboratory evaluation of imidacloprid against *Microtermes obesi* (Holmgren)(Isoptera: Macrotermitinae). Proceedings of the Pakistan Academy of Sciences, 51, 43–48.

[ins12726-bib-0080] Mo, J.C. , Guo, J.Q. and Gong, Y.G. (2008) Control of Termites in Urban and Rural Areas. Chemical Industry Press, Beijing, 112 pp. (in Chinese).

[ins12726-bib-0081] MRP (2010) Maxwell Robinson Phelps Termite Report. Maxwell, Robinson and Phelps. http://www.pestcontrolperth.com/wpcontent/uploads/2010/06/Maxwell-RobinsonPhelps-MRP-TermiteReport.pdf.

[ins12726-bib-0082] Murugan, K. and Vasugi, C. (2011) Combined effect of Azadirachta indica and the entomopathogenic nematode Steinernema glaseri against subterranean termite, *Reticulitermes flavipes* . Journal of Entomological and Acarological Research, 43, 253–259.

[ins12726-bib-0083] Natsir, H. and Dali, S. (2014) Production and application of chitin deacetylase from *Bacillus licheniformis* HSA3‐1a as biotermicide. Marina Chimica Acta, 15, 1–12.

[ins12726-bib-0084] Negassa, W. and Sileshi, G.W. (2018) Integrated soil fertility management reduces termite damage to crops on degraded soils in western Ethiopia. Agriculture, Ecosystems & Environment, 251, 124–131.

[ins12726-bib-0085] Nyagumbo, I. , Munamati, M. , Mutsamba, E.F. , Thierfelder, C. , Cumbane, A. and Dias, D. (2015) The effects of tillage, mulching and termite control strategies on termite activity and maize yield under conservation agriculture in Mozambique. Crop Protection, 78, 54–62.

[ins12726-bib-0086] Oberst, S. , Bann, G. , Lai, J.C. and Evans, T.A. (2017) Cryptic termites avoid predatory ants by eavesdropping on vibrational cues from their footsteps. Ecology Letters, 20, 212–221. 28111901 10.1111/ele.12727

[ins12726-bib-0087] Omoya, F. and Kelly, B. (2014) Variability of the potency of some selected entomopathogenic bacteria (*Bacillus* spp. and *Serratia* spp.) on termites, *Macrotermes bellicosus* (Isoptera: Termitidae) after exposure to magnetic fields. International Journal of Tropical Insect Science, 34, 98–105.

[ins12726-bib-0088] Pandey, A. , Chattopadhyay, P. , Banerjee, S. , Pakshirajan, K. and Singh, L. (2012) Antitermitic activity of plant essential oils and their major constituents against termite *Odontotermes assamensis* Holmgren (Isoptera: Termitidae) of North East India. International Biodeterioration & Biodegradation, 75, 63–67.

[ins12726-bib-0089] Pandey, P. , Singha, L.P. and Singha, B. (2013) Colonization and antagonistic activity of entomopathogenic *Aspergillus* sp. against tea termite (*Microcerotermes beesoni* Snyder). Current Science, 105, 1216–1219.

[ins12726-bib-0090] Paul, B. , Khan, M.A. , Paul, S. , Shankarganesh, K. and Chakravorty, S. (2018) Termites and Indian agriculture Termites and Sustainable Management (eds. Cham Md. Aslam, Khan Wasim Ahmad), pp. 51–96. Springer, Saudi Arabia.

[ins12726-bib-0091] Petráková, L. , Líznarová, E. , Pekár, S. , Haddad, C.R. , Sentenská, L. and Symondson, W.O. (2015) Discovery of a monophagous true predator, a specialist termite‐eating spider (Araneae: Ammoxenidae). Scientific Reports, 5, 14013. 26359085 10.1038/srep14013 PMC4566138

[ins12726-bib-0092] Poulsen, M. (2015) Towards an integrated understanding of the consequences of fungus domestication on the fungus‐growing termite gut microbiota. Environmental Microbiology, 17, 2562–2572. 25581852 10.1111/1462-2920.12765

[ins12726-bib-0093] Poulsen, M. , Hu, H. , Li, C. , Chen, Z. , Xu, L. , Otani, S . *et al* (2014) Complementary symbiont contributions to plant decomposition in a fungus‐farming termite. Proceedings of the National Academy of Sciences USA, 111, 14500–14505. 10.1073/pnas.1319718111 PMC4209977 25246537

[ins12726-bib-0094] Qureshi, N.A. , Ashraf, A. , Afzal, M. , Ullah, N. , Iqbal, A. and Haleem, S. (2015) Toxic potential of *Melia azedarach* leaves extract against *Odontotermes obesus* and *Microtermes obesi* . International Journal of Biosciences, 6, 120–127.

[ins12726-bib-0095] Rana, B. and Dinesh, K. (2014) Study of bio‐efficacy of entomopahogenic fungi for suppression of termite incidence in maiz. International Journal of Plant Protection, 7, 377–381.

[ins12726-bib-0096] Rathour, K.S. , Sudershan, G. , Das, T. , Pargat, S. , Anjani, K. and Somvanshi, V.S. (2014) Biological management of subterranean termites (*Odontotermes obesus*) infesting wheat and pearl millet crops by entomopathogenic nematodes. Indian Journal of Nematology, 44, 97–100.

[ins12726-bib-0096a] Ravan, S. , Khan, I.A. , Manzoor, F. and Khan, Z. (2015) Feeding habitats and wood preferences of termites in Iran. Journal of Entomology and Zoology Studies, 3, 20–23.

[ins12726-bib-0097] Ravindran, K. , Qiu, D. and Sivaramakrishnan, S. (2015) Sporulation characteristics and virulence of *Metarhizium anisopliae* against subterranean termites (*Coptotermes formosanus*). International Journal of Microbiological Research, 6, 01–04.

[ins12726-bib-0098] Rust, M.K. and Su, N.Y. (2012) Managing social insects of urban importance. Annual Review of Entomology, 57, 355–375. 10.1146/annurev-ento-120710-100634 21942844

[ins12726-bib-0099] Saljoqi, A.U.R. , Muhammad, N. , Khan, I.A. , Nadeem, M. and Salim, M. (2014) Effect of different insecticides against termites, *Heterotermes indicola* L.(Isoptera: Termitidae) as slow acting toxicants. Sarhad Journal of Agriculture, 30.

[ins12726-bib-0100] Samsuddin, A.S. , Sajap, A.S. and Mohamed, R. (2016) *Metarhizium anisopliae* of peninsular Malaysia origin poses high pathogenicity toward *Coptotermes curvignathus*, a major wood and tree pest. The Malaysian Forester, 78, 41–48.

[ins12726-bib-0101] Sane, C.A.B. , Rouland‐Lefevre, C. , Grechi, I. , Rey, J.Y. , Vayssieres, J.F. , Diame, L . *et al* (2016) Diversité, nuisances et modes de gestion des termites (Isoptera) dans les agrosystèmes sénégalais. International Journal of Biological and Chemical Sciences, 10, 134–154.

[ins12726-bib-0102] Seo, S.M. , Kim, J. , Kang, J. , Koh, S.H. , Ahn, Y.J. , Kang, K.S . *et al* (2014) Fumigant toxicity and acetylcholinesterase inhibitory activity of 4 Asteraceae plant essential oils and their constituents against Japanese termite (*Reticulitermes speratus* Kolbe). Pesticide Biochemistry and Physiology, 113, 55–61. 25052527 10.1016/j.pestbp.2014.06.001

[ins12726-bib-0103] Shahina, F. and Tabassum, K. (2010) Virulence of *Steinernema pakistanense* against different insect species in laboratory condition. Pakistan Journal of Nematology, 28, 279–284.

[ins12726-bib-0104] Shahina, F. , Tabassum, K. , Salma, J. and Mahreen, G. (2011) Biopesticidal affect of *Photorhabdus luminescens* against *Galleria mellonella* larvae and subteranean termite (Termitidae: Macrotermis). Pakistan Journal of Nematology, 29, 35–43.

[ins12726-bib-0105] Sharma, S. , Verma, M. and Sharma, A. (2013) Utilization of non edible oil seed cakes as substrate for growth of *Paecilomyces lilacinus* and as biopesticide against termites. Waste and Biomass Valorization, 4, 325–330.

[ins12726-bib-0106] Sileshi, A. , Sori, W. and M., D. (2013) Laboratory evaluation of entomopathogenic fungi *Metarhizium anisophilae* and *Beauveria bassiana* against termite, *Macrotermes* (Isoptera: Termitidae). Asian Journal of Plant Sciences, 12, 1–10.

[ins12726-bib-0107] Singha, D. , Singha, B. and Dutta, B. (2010) *In vitro* pathogenicity of *Bacillus thuringiensis* against tea termites. Journal of Biological Control, 24, 279–281.

[ins12726-bib-0108] Staunton, I. (2012) *Termite Cost: The Real Story About Termite Damage in Australia* . https://termikill.com.au/termite-cost/.

[ins12726-bib-0109] Subekti, N. , Yoshimura, T. , Rokhman, F. and Mastur, Z. (2015) Potential for subterranean termite attack against five bamboo speciesin correlation with chemical components. Procedia Environmental Sciences, 28, 783–788.

[ins12726-bib-0110] Tahseen, Q. , Akram, M. , Mustaqim, M. and Ahlawat, S. (2014) Descriptions of *Pelodera scrofulata* sp. nov. and *Pelodera aligarhensis* sp. nov. (Nematoda: Rhabditidae) with supplementary information on *Pelodera teres* (Schneider, 1866). Journal of Natural History, 48, 1027–1053.

[ins12726-bib-0111] Tasida, J. and Gobena, T. (2013) Evaluation of chemical, botanical and cultural managements of termites control. Journal of World Applied Sciences, 22, 583–588.

[ins12726-bib-0112] Tian, W.J. , Ke, Y.L. , Zhuang, T.Y. , Wang, C.X. , Li, M. , Liu, R.Q . *et al* (2009) Incipient colony development and biology of *Odontotermes formosanus* (Shiraki) and *O. hainanensis* (Light)(Isoptera: Termitidae). Journal of Agricultural and Urban Entomology, 26, 147–157.

[ins12726-bib-0113] Traoré, S. , Tigabu, M. , Jouquet, P. , Ouédraogo, S.J. , Guinko, S. and Lepage, M. (2015) Long‐term effects of *Macrotermes* termites, herbivores and annual early fire on woody undergrowth community in Sudanian woodland, Burkina Faso. Flora‐Morphology, Distribution, Functional Ecology of Plants, 211, 40–50.

[ins12726-bib-0114] Tsunoda, K. and Yoshumura, T. (2004) Current termite management in Japan. *Proceedings of 1st Pacific‐Rim Termite Research Group*. 8–9 March 2004, Penang, Malaysia. pp. 1–5.

[ins12726-bib-0115] van Huis, A. (2017) Cultural significance of termites in sub‐Saharan Africa. Journal of Ethnobiology and Ethnomedicine, 13, 1–12. 28126033 10.1186/s13002-017-0137-z PMC5270236

[ins12726-bib-0116] Verma, M. , Sharma, S. and Prasad, R. (2009) Biological alternatives for termite control: a review. International Biodeterioration & Biodegradation, 63, 959–972.

[ins12726-bib-0117] Verma, M. , Verma, S. and Sharma, S. (2018) Eco‐friendly termite management in tropical conditions Termites and Sustainable Management. pp. 137–164. Springer, Cham.

[ins12726-bib-0118] Verma, S. , Sharma, S. and Malik, A. (2016) Termiticidal and repellency efficacy of botanicals against *Odontotermes obesus* . International Journal of Research in Biosciences, 5, 52–59.

[ins12726-bib-0119] Verma, S. , Verma, M. , Sharma, S. and Malik, A. (2013) Determination of phytocomponents of *Jatropha curcas* root by GC‐MS analysis and their termiticidal activity. International Journal of Ecology and Environmental Sciences, 39, 159–169.

[ins12726-bib-0120] Vongkaluang, C. (2004) Current termite management in Thailand. *Proceedings of 1st Pacific‐Rim Termite Research Group* ; 8–9 March 2004, Penang, Malaysia. pp. 43–46.

[ins12726-bib-0121] Wagutu, G. (2017) Efficacy of entomopathogenic nematode (*Steinernema karii*) in control of termites (*Coptotermes formosanus*). Journal of Agriculture, Science and Technology, 18, 55–64.

[ins12726-bib-0122] Wang, C. and Henderson, G. (2013) Evidence of Formosan subterranean termite group size and associated bacteria in the suppression of entomopathogenic bacteria, *Bacillus thuringiensis* subspecies *israelensis* and *thuringiensis* . Annals of the Entomological Society of America, 106, 454–462.

[ins12726-bib-0123] Wang, C. , Henderson, G. , Gautam, B.K. and Chen, X. (2014) Lethal and sublethal effects of lufenuron on the Formosan subterranean termite (Isoptera: Rhinotermitidae). Journal of Economic Entomology, 107, 1573–1581. 25195450 10.1603/ec14103

[ins12726-bib-0124] Wright, M.S. and Lax, A.R. (2013) Combined effect of microbial and chemical control agents on subterranean termites. Journal of Microbiology, 51, 578–583. 10.1007/s12275-013-2628-5 24037651

[ins12726-bib-0125] Wu, W. , Gu, D. , Yan, S. and Li, Z. (2019) RNA interference of endoglucanases in the formosan subterranean termite *Coptotermes formosanus* Shiraki (Blattodea: Rhinotermitidae) by dsRNA injection or ingestion. Journal of Insect Physiology, 112, 15–22. 30472007 10.1016/j.jinsphys.2018.11.007

[ins12726-bib-0126] Wu, W. and Li, Z. (2018) dsRNA injection successfully inhibited two endogenous β‐glucosidases in *Coptotermes formosanus* (Isoptera: Rhinotermitidae). Journal of Economic Entomology, 111, 860–867. 29360999 10.1093/jee/tox371

[ins12726-bib-0127] Xie, Y. , Wang, K. , Huang, Q. and Lei, C. (2014) Evaluation toxicity of monoterpenes to subterranean termite, *Reticulitermes chinensis* Snyder. Industrial Crops and Products, 53, 163–166.

[ins12726-bib-0128] Yadav, A.N. , Sachan, S.G. , Verma, P. , Kaushik, R. and Saxena, A.K. (2016) Cold active hydrolytic enzymes production by psychrotrophic Bacilli isolated from three sub‐glacial lakes of NW Indian Himalayas. Journal of Basic Microbiology, 56, 294–307. 26933936 10.1002/jobm.201500230

[ins12726-bib-0129] Yanagawa, A. , Fujiwara‐Tsujii, N. , Akino, T. , Yoshimura, T. , Yanagawa, T. and Shimizu, S. (2012) Odor aversion and pathogen‐removal efficiency in grooming behavior of the termite *Coptotermes formosanus* . PLoS ONE, 7, e47412. 23077609 10.1371/journal.pone.0047412 PMC3471821

[ins12726-bib-0130] Yanagawa, A. , Yokohari, F. and Shimizu, S. (2009) The role of antennae in removing entomopathogenic fungi from cuticle of the termite, Coptotermes formosanus. Journal of Insect Science, 9, 10.1673/031.009.0601. PMC3011873 19611249

[ins12726-bib-0131] Yeoh, B.H. and Lee, C.Y. (2007) Tunneling activity, wood consumption and survivorship of *Coptotermes gestroi*, *Coptotermes curvignathus* and *Coptotermes kalshoveni* (Isoptera: Rhinotermitidae) in the laboratory. Sociobiology, 50, 1087–1096.

[ins12726-bib-0132] Yii, J.E. , Bong, C.F.J. , King, J.H.P. and Kadir, J. (2015) Synergism of entomopathogenic fungus, *Metarhizium anisopliae* incorporated with fipronil against oil palm pest subterranean termite, Coptotermes curvignathus. Plant Protection Science, 52, 35–44.

[ins12726-bib-0133] Yu, H. , Gouge, D.H. and Shapiro‐Ilan, D.I. (2010) A novel strain of *Steinernema riobrave* (Rhabditida: Steinernematidae) possesses superior virulence to subterranean termites (Isoptera: Rhinotermitidae). Journal of Nematology, 42, 91–95. 22736844 PMC3380470

[ins12726-bib-0134] Yuan, Z. and Hu, X.P. (2012) Repellent, antifeedant, and toxic activities of *Lantana camara* leaf extract against *Reticulitermes flavipes* (Isoptera: Rhinotermitidae). Journal of Economic Entomology, 105, 2115–2121. 23356077 10.1603/ec12026

[ins12726-bib-0135] Zadji, L. , Baimey, H. , Afouda, L. , Moens, M. and Decraemer, W. (2014a) Characterization of biocontrol traits of heterorhabditid entomopathogenic nematode isolates from South Benin targeting the termite pest *Macrotermes bellicosus* . BioControl, 59, 333–344.

[ins12726-bib-0136] Zadji, L. , Baimey, H. , Afouda, L. , Moens, M. and Decraemer, W. (2014b) Effectiveness of different *Heterorhabditis* isolates from Southern Benin for biocontrol of the subterranean termite, *Macrotermes bellicosus* (Isoptera: Macrotermitinae), in laboratory trials. Nematology,16, 109–120.

[ins12726-bib-0137] Zamoum, M. , Berchiche, S. , Sai, K. , Triggia, O. and Tarasco, E. (2011) Preliminary survey of the occurrence of entomopathogenic nematodes and fungi in the forest soils of Algeria. Silva Lusitana, 19, 143–147.

[ins12726-bib-0138] Zhang, M. and Govindaraju, M. (2018) Sugarcane production in China. Sugarcane: Technology and Research (ed. De Oliveira A.), pp. 49–66.

[ins12726-bib-0139] Zhang, P.B. , Yan, X. , Qiu, X.H. and Han, R.C. (2010) Application of transgenic *Enterobacter cloacae* with the insecticidal tcdA 1 B 1 genes for control of *Coptotermes formosanus* (Isoptera: Rhinotermitidae) in the field. Sociobiology, 56, 27–38.

[ins12726-bib-0140] Zhang, S. and Mo, J.C. (2014) Effect of *Autographa californica* nuclear polyhedrosis virus suspension concentrate against the workers of *Coptotermes formosanus* Shiraki. Forest Pest and Disease, 3, 18–21.

[ins12726-bib-0141] Zhong, J. and Liu, L. (2002) Termite fauna in China and their economic importance. Sociobiology, 40, 25–32.

[ins12726-bib-0142] Zhong, J.H. and Li, B.R. (2004) Current termite management in China. *Proceedings of 1st Pacific‐Rim Termite Research Group*. 8–9 March 2004, Penang, Malaysia. pp. 11–16.

[ins12726-bib-0143] Zhou, X. , Oi, F.M. and Scharf, M.E. (2006) Social exploitation of hexamerin: RNAi reveals a major caste‐regulatory factor in termites. Proceedings of the National Academy of Sciences USA, 103, 4499–4504. 10.1073/pnas.0508866103 PMC1450200 16537425

[ins12726-bib-0144] Zhou, X. , Wheeler, M.M. , Oi, F.M. and Scharf, M.E. (2008a) Inhibition of termite cellulases by carbohydrate‐based cellulase inhibitors: Evidence from *in vitro* biochemistry and *in vivo* feeding studies. Pesticide Biochemistry and Physiology, 90, 31–41.

[ins12726-bib-0145] Zhou, X. , Wheeler, M.M. , Oi, F.M. and Scharf, M.E. (2008b) RNA interference in the termite *Reticulitermes flavipes* through ingestion of double‐stranded RNA. Insect Biochemistry and Molecular Biology, 38, 805–815. 18625404 10.1016/j.ibmb.2008.05.005

